# Potential Therapeutic Targets for Androgenetic Alopecia (AGA) in Obese Individuals as Revealed by a Gut Microbiome Analysis: A Mendelian Randomization Study

**DOI:** 10.3390/nu17111892

**Published:** 2025-05-31

**Authors:** Yongwei Li, Xi Liao, Siwen Tang, Qian Wang, Heng Lin, Xi Yu, Ying Xiao, Xiaoyu Tao, Tian Zhong

**Affiliations:** Faculty of Medicine, Macau University of Science and Technology, Taipa, Macao 999078, China

**Keywords:** gut microbiome, androgenetic alopecia (AGA), obesity, Mendelian randomization (MR)

## Abstract

**Objective:** This study aimed to investigate the role of the gut microbiome in androgenetic alopecia (AGA) among obese individuals using Mendelian randomization (MR), and to identify potential therapeutic targets for mitigating AGA in this population. **Methods:** Genomic data for 412 gut microbiomes, AGA, and obesity were obtained from genome-wide association studies (GWAS). Bidirectional MR was performed using inverse variance weighted (IVW) as the primary analysis method, complemented by sensitivity analyses. Potential therapeutic targets within the gut microbiome associated with AGA in obese individuals were identified. **Results:** Two gut microbiomes were identified as having a significant impact on obese individuals with AGA. Specifically, the abundance of the sulfoglycolysis pathway in gut bacteria was found to significantly increase the risk of both obesity and AGA. In contrast, the abundance of the de novo biosynthesis of the adenosine ribonucleotide pathway in gut bacteria was associated with a significant increase in the risk of obesity but a significant decrease in the risk of AGA. **Conclusions:** The abundance of gut bacterial pathways, including sulfoglycolysis and the de novo biosynthesis of adenosine ribonucleotides, can serve as potential therapeutic targets for managing obesity-associated AGA. These findings offer a novel research direction for the development of innovative diagnostic and treatment strategies for patients with obesity and AGA.

## 1. Introduction

Androgenetic alopecia (AGA) is the most prevalent form of progressive hair loss globally [1], with an estimated prevalence range of 0.2% to 2% in the global population [2,3,4]. In certain developed European countries, the incidence of AGA among men aged 50 and older can reach up to 50%, increasing progressively with age [5]. Currently, in Europe, the annual direct medical expenses for AGA patients may amount to several hundred euros per individual [6]. Moreover, indirect costs, including productivity losses and psychological treatment expenses, significantly contribute to economic burdens at both individual and societal levels. Notably, the incidence of AGA is markedly higher among obese individuals compared to the general population, with a significant positive correlation observed between AGA severity and obesity levels [7]. Consequently, some studies have proposed that obesity may represent a potential risk factor for AGA [8]. However, this hypothesis remains to be fully validated by the scientific community and requires further comprehensive investigation.

The intestine, as a primary site of digestion in the human body, not only harbors diverse gut microbiota but also encompasses multiple unique gut bacterial pathways, collectively referred to as the gut microbiome. Recent studies have demonstrated that the gut microbiome is associated with both obesity and AGA. The translocation of intestinal microorganisms into host signaling pathways (the molecular and cellular biological mechanisms underlying host functions, including food intake behavior, energy acquisition, energy expenditure, and fat storage) can induce obesity [9,10], while dysregulation of the gut microbiota–gut–brain axis, including immune responses, energy metabolism, hormone secretion, and nutrient metabolism, can contribute to AGA [11,12].

Therefore, to investigate the relationships among the gut microbiome, obesity and AGA, this study employed a bidirectional Mendelian randomization (MR) analysis using existing genome-wide association study (GWAS) data [13]. The aim was to identify potential therapeutic targets within the gut microbiome for individuals with obesity-associated AGA and to provide new treatment strategies for this population.

## 2. Methods

### 2.1. Data

The study utilized summary data derived from the gut microbiome obtained from the GWAS of the Dutch Microbiome Project. This dataset comprises information on 7738 European participants and encompasses 207 gut microbiota abundances and 205 gut bacterial pathway abundances, collectively representing the microbial composition (accessible for download at https://www.ebi.ac.uk/gwas/studies/understudy (accessed on 1 Octorber 2024), accession numbers GCST90027446-GCST90027857).

The study utilized GWAS data for AGA, which encompass 207,036 European individuals, including 66,172 cases and 140,864 controls (available for download at https://www.ebi.ac.uk/gwas/studies/understudy (accessed on 1 Octorber 2024), accession number GCST90043616).

The study utilized GWAS data for obesity, which encompass 456,348 European individuals, including 581 cases and 455,767 controls (available for download at https://www.ebi.ac.uk/gwas/studies/understudy (accessed on 1 Octorber 2024), accession number GCST90043672).

### 2.2. Statistics

Through a bidirectional MR analysis, we evaluated the causal relationships among the gut microbiome, obesity, and AGA. Specifically, we conducted three analyses: (1) evaluating the causal effect of the gut microbiome on AGA by designating the gut microbiome as exposure 1 and AGA as the outcome; (2) assessing the impact of the specific gut microbiome significantly associated with AGA on obesity by using these specific gut microbiomes as exposure 3 and obesity as the outcome; (3) investigating the influence of obesity on AGA by setting obesity as exposure 2 and AGA as the outcome.

**Figure 1:**
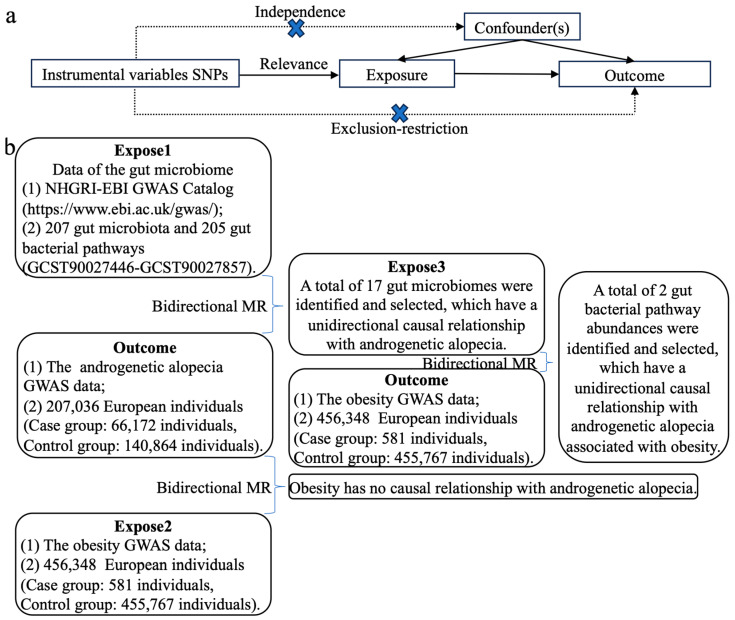
(**a**) Flowchart of the study design. (**b**) An overview of the study design aimed at identifying gut microbiome intervention targets for obesity-associated AGA.

Three crucial assumptions must be fulfilled for a valid MR analysis [14]:

(1) Relevance assumption: Single-nucleotide polymorphisms (SNPs) must exhibit a strong association with the exposure. This association is quantified via the F statistic, where an F > 10 indicates strong relevance (formula: F = β^2^/Se^2^)
(2) Independence assumption: The SNPs should not be associated with any confounding factors that may influence both the exposure and the outcome, nor should they be directly associated with the outcome.
(3) Exclusion–restriction assumption: SNPs should influence the outcome solely through the exposure rather than via alternative pathways. This implies that there should be no horizontal pleiotropy (Figure 1a) [11,12].

### 2.3. Selection of Genetic Instrumental Variables (IVs)

To ensure the validity and accuracy of the study, we applied the following criteria to select optimal IVs (Figure 1b):

(1) To acquire more extensive data, SNPs from the GWAS data on the exposure were selected based on their strong association with the exposure; specifically, SNPs with *p*-values < 0.00001.
(2) Based on the independence assumption of MR, each SNP must be independent of the others. To ensure that there was no linkage disequilibrium (LD) among the SNPs, we applied an LD threshold of R^2^ = 0.001 and a distance criterion of ≤10,000 kb.
(3) To ensure that the same SNPs have identical alleles in both the exposure and the outcome, ambiguous alleles were excluded.
(4) The exclusion–restriction assumption requires that SNPs associated with the exposure should not be directly related to the outcome. Consequently, the online tool National Institutes of Health *LDlink* (https://ldlink.nih.gov/?tab=ldtrait#ldtrait-tab (accessed on 1 November 2024)) was utilized to identify traits directly associated with the SNPs. SNPs linked to the outcome were excluded, and SNPs lacking alternative loci were removed [14].
(5) The sensitivity analysis removed SNPs with potential pleiotropy.

### 2.4. Statistical Analysis

The IVW method was employed as the principal approach to efficiently and accurately estimate the unbiased causal effect between the exposure and the outcome, under the assumption that there is no horizontal pleiotropy or heterogeneity among all SNPs. The MR–Egger intercept from the MR–Egger test was used to evaluate the presence of horizontal pleiotropy, thereby detecting the influence of pleiotropy on the overall model. Cochran’s Q test was utilized to assess heterogeneity among SNPs. Additionally, the “leave-one-out” approach was adopted to conduct a stability analysis, evaluating the impacts of individual SNPs on the causal association. All bidirectional MR analyses were performed using R software (version 4.4.0).

## 3. Results

### 3.1. The Association Between the Gut Microbiome (Exposure 1) and AGA

**Figure 2:**
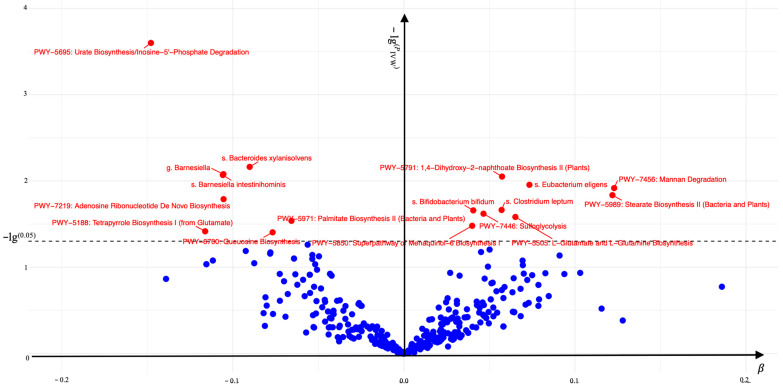
Associations of 17 gut microbiomes with AGA. The associations that remained significant after multiple testing corrections are labeled in the volcano plot. Red dots above the dashed line indicate statistically significant associations.

**Figure 3:**
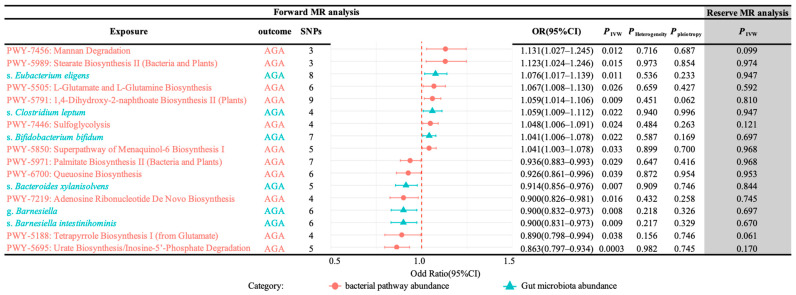
Forest plot of MR estimates between the gut microbiome and AGA. This figure displays the IVW estimates for gut microbiomes significantly associated with AGA. Red dots and green triangles represent the IVW estimates, while solid lines indicate the 95% confidence intervals of these estimates. An OR > 1 indicates an increased risk, whereas an OR < 1 indicates a decreased risk. *P*_Heterogeneity_ denotes the *p*-value for heterogeneity and *P*_pleiotropy_ denotes the *p*-value for horizontal pleiotropy; *p*-value > 0.05 indicates non-significance (OR, odds ratio).

**Figure 4:**
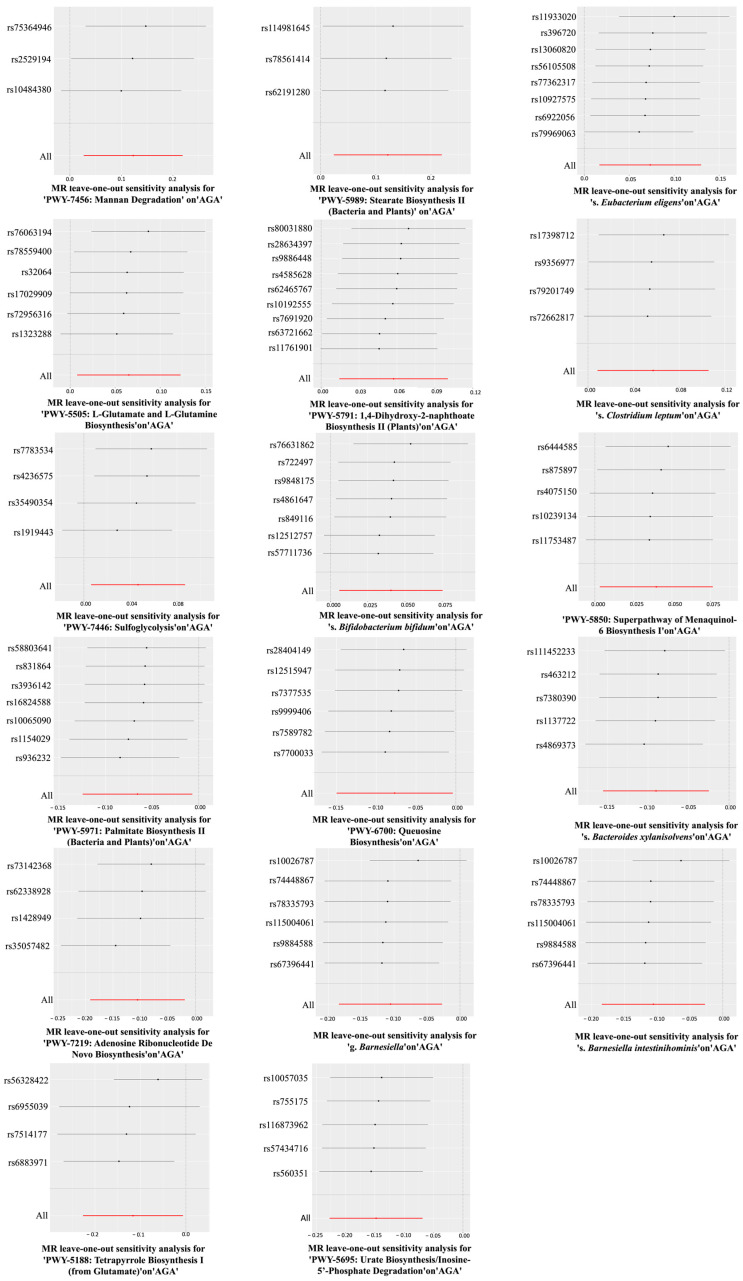
Leave-one-out analysis diagrams for causal effects of the gut microbiome on AGA.

A total of 17 gut microbiomes were identified and selected (Figure 2). In this study, the IVW method served as the primary analytical approach. The application of this method revealed no evidence of horizontal pleiotropy (*P*_pleiotropy_ > 0.05) or heterogeneity (*P*_Heterogeneity_ > 0.05) (Figure 3). Additionally, the consistent and robust results from the “leave-one-out” sensitivity analysis significantly enhances the credibility of the causal inference (Figure 4). These findings suggest that even if individual IVs may have limitations, the overall causal inference remains reliable.

The gut bacterial pathways of mannan degradation (IVW, OR (95% CI) = 1.027–1.245, *P*_IVW_ = 0.012), stearate biosynthesis II (bacteria and plants) (IVW, OR (95% CI) = 1.024–1.246, *P*_IVW_ = 0.015), L-glutamate and L-glutamine biosynthesis (IVW, OR (95% CI) = 1.008–1.130, *P*_IVW_ = 0.026), 1,4-dihydroxy-2-naphthoate biosynthesis II (plants) (IVW, OR (95% CI) = 1.014–1.106, *P*_IVW_ = 0.009), sulfoglycolysis (IVW, OR (95% CI) = 1.006–1.091, *P*_IVW_ = 0.024), the superpathway of menaquinol-6 biosynthesis I (IVW, OR (95% CI) = 1.003–1.078, *P*_IVW_ = 0.033) and the gut microbiota of s. *Eubacterium eligens* (IVW, OR (95% CI) = 1.017–1.139, *P*_IVW_ = 0.011), s. *Clostridium leptum* (IVW, OR (95% CI) = 1.009–1.112, *P*_IVW_ = 0.022), and s. *Bifidobacterium bifidum* (IVW, OR (95% CI) = 1.006–1.078, *P*_IVW_ = 0.022) were associated with a significant increase in the risk of AGA.

Conversely, the gut bacterial pathways of palmitate biosynthesis II (bacteria and plants) (IVW, OR (95% CI) = 0.883–0.993, *P*_IVW_ = 0.029), queuosine biosynthesis (IVW, OR (95% CI) = 0.861–0.996, *P*_IVW_ = 0.039), adenosine ribonucleotide de novo biosynthesis (IVW,OR (95% CI) = 0.826–0.981, *P*_IVW_ = 0.016), tetrapyrrole biosynthesis I (from glutamate) (IVW,OR (95% CI) = 0.798–0.994, *P*_IVW_ = 0.038), urate biosynthesis/inosine-5′-phosphate degradation (IVW,OR (95% CI) = 0.797–0.934, *P*_IVW_ = 0.0003) and the gut microbiota of s. *Bacteroides xylanisolvens* (IVW, OR (95% CI) = 0.856–0.976, *P*_IVW_ = 0.007), g. *Barnesiella* (IVW, OR (95% CI) = 0.832–0.973, *P*_IVW_ = 0.008), and s. *Barnesiella intestinihominis* (IVW, OR (95% CI) = 0.831–0.973, *P*_IVW_ = 0.009) were found to significantly decrease the risk of AGA. These associations were statistically significant in the IVW analysis (*P*_IVW_ < 0.05).

MR was employed to assess whether the aforementioned gut microbiome, including 11 gut bacterial pathway abundances and 6 gut microbiota abundances, was influenced by AGA. The reverse MR analysis did not reveal any significant causal associations (reverse MR *P*_IVW_ > 0.05, Figure 3).

### 3.2. The Association Between the Gut Microbiomes (Exposure 3) and Obesity

**Figure 5:**
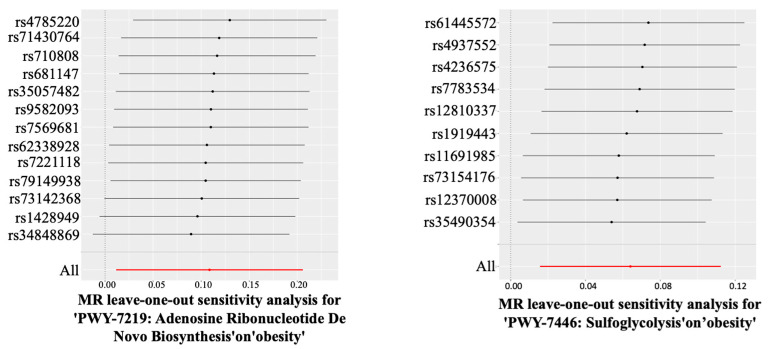
Leave-one-out analysis diagrams for causal effects of the gut microbiome on obesity-associated AGA.

Among the 17 gut microbiomes, only two gut bacterial pathway abundances were significantly associated with obesity. The pathways of adenosine ribonucleotide de novo biosynthesis (IVW, OR (95% CI) = 1.012–1.229, *P*_IVW_ = 0.028) and sulfoglycolysis (IVW, OR (95% CI) = 1.016–1.119, *P*_IVW_ = 0.009) were found to significantly increase the risk of obesity. Notably, there was no evidence of horizontal pleiotropy (*P*_pleiotropy_ > 0.05) or heterogeneity (*P*_Heterogeneity_ > 0.05) (Table 1). Furthermore, the consistent and robust results from the “leave-one-out” sensitivity analysis substantially enhance the credibility of the causal inference (Figure 5).

A reverse MR analysis was employed to assess whether obesity influences the 17 gut microbiomes. The results did not indicate any causality (reverse MR, *P*_IVW_ > 0.05, Table 1).

### 3.3. The Association Between Obesity (Exposure 2) and AGA

The bidirectional MR analysis of the association between AGA and obesity did not find any causal relationship (bidirectional MR analysis, *P*_IVW_ > 0.05, Table 2).

## 4. Discussion

The high incidence of AGA in obese individuals may be attributed to the inflammatory signals induced by obesity, which can lead to the depletion of hair follicle stem cells (HFSCs) and inhibit hair follicle regeneration [15]. Furthermore, as an essential external feature contributing to personal image, hair loss may result in mental health issues such as reduced self-esteem, anxiety, and depression, significantly impacting quality of life. This psychological burden may drive individuals toward more reclusive behavior, accompanied by overeating and physical inactivity, further exacerbating obesity and creating a vicious cycle that increases the risk of AGA in obese individuals [16]. These findings suggest that obesity is not a direct causative factor for AGA, aligning with the conclusions of this study. However, from the perspective of the gut microbiome, both obesity and AGA may be influenced by specific metabolic pathways of gut bacteria. Specifically, the activation of the gut bacterial pathway of sulfoglycolysis promotes both obesity and AGA, indicating the potential involvement of a common molecular mechanism in their pathological processes. Conversely, while the gut bacterial pathway of adenosine ribonucleotide de novo biosynthesis does not improve obesity, it demonstrates a protective effect on AGA. Despite the dual effects of this metabolic pathway, the role of the gut microbiota in both conditions cannot be overlooked. Therefore, exploring the mechanisms underlying the gut microbiota may offer novel therapeutic strategies for addressing AGA in obese individuals.

### 4.1. The Impact of the Gut Bacterial Pathway of Sulfoglycolysis on Obesity and AGA

The gut bacterial sulfoglycolysis pathway is involved in the generation of energy molecules, such as through the degradation of sulfoglycolipids to sustain energy metabolism [17]. During this process, the elevated cellular demand for antioxidants necessitates an increased requirement for nicotinamide adenine dinucleotide phosphate (reduced form) (NAD(P)H). NAD(P)H plays a pivotal role in the regeneration of glutathione (GSH), thereby ensuring the maintenance of reduced GSH levels [18]. This redox imbalance not only compromises physiological functions including energy metabolism, fat storage, and insulin sensitivity but also profoundly affects the pathogenesis of AGA via the metabolic products generated during the process.

NAD(P)H plays a crucial role in both fat synthesis and energy metabolism. During fat synthesis, NAD(P)H facilitates the conversion of acetyl-CoA to malonyl-CoA, which is subsequently elongated into palmitic acid [19]. Stearoyl-CoA desaturase 1 (Scd1) then converts saturated fatty acids into monounsaturated fatty acids. The inhibition of fatty acid oxidation by the antioxidant system promotes continuous fatty acid synthesis, leading to fat accumulation. In energy metabolism, NAD(P)H is consumed by NAD(P)H oxidase (NOX) to generate reactive oxygen species (ROS), which inhibit insulin secretion and disrupt glucose and lipid homeostasis, contributing to obesity [18]. Notably, acetyl-CoA, a key intermediate in fat synthesis, not only significantly influences fatty acid synthesis but also triggers AGA by shortening the anagen phase of hair follicles [20,21].

GSH plays a pivotal role in the antioxidant defense system by donating hydrogen atoms to neutralize reactive oxygen species (ROS), resulting in the accumulation of its oxidized form, glutathione disulfide (GSSG), within the body [22]. While this mechanism may offer potential benefits for AGA improvement, the metabolism of GSH is closely linked to sex hormone regulation [23]. Specifically, GSH can upregulate the levels of sex-hormone-binding globulin (SHBG) [24], which may suppress hair follicle activity and thereby contribute to AGA development. Moreover, GSH influences obesity progression indirectly by modulating satiety and appetite signals in the hypothalamus [17,25,26].

Therefore, the gut bacterial pathway of sulfoglycolysis not only disrupts cellular redox homeostasis and affects fatty acid synthesis and energy metabolism, thereby influencing obesity, but also produces intermediate products that significantly impact the development of AGA. This suggests that the sulfoglycolysis pathway shares common mechanistic features with both obesity and AGA. Inhibiting this pathway can mitigate the progression of obesity and simultaneously ameliorate the advancement of AGA.

### 4.2. The Impact of the Gut Bacterial Pathway of Adenosine Ribonucleotide De Novo Biosynthesis on Obesity and AGA

Adenosine ribonucleotides, through the fermentation of pyruvate, generate lactic acid, a glycolytic byproduct [27]. Lactic acid serves not only as a primary fuel for the tricarboxylic acid (TCA) cycle and a precursor for gluconeogenesis but can also be converted into short-chain fatty acids under various conditions [28,29], contributing to obesity. Furthermore, lactic acid binds to the cell surface receptor G protein-coupled receptor 81 (GPR81), regulating insulin expression and inhibiting lipolysis [30,31]. This interaction recruits macrophages, promoting inflammatory responses that lead to insulin resistance [32], ultimately resulting in obesity.

Adenosine ribonucleotides, such as ATP and ADP, are essential components in the synthesis of DNA and RNA. Fluctuations in their levels directly influence cellular metabolic processes and play a crucial role in intracellular signal transduction [33]. Under the influence of androgens such as dihydrotestosterone (DHT), an energy metabolism imbalance in hair follicles can lead to follicular atrophy. However, K^ATP^ channels, by sensing ATP and ADP levels, can mitigate this metabolic stress, protecting hair follicle cells from the adverse effects of androgens. By modulating the electrophysiological activity of hair follicle cells, K^ATP^ channels also enhance the metabolic state of the follicles, thereby prolonging the anagen phase and promoting hair regrowth [34]. Additionally, adenosine nucleotides can bind with pyruvate to reduce its conversion to acetyl-CoA [24], thereby inhibiting excessive androgen production and improving AGA.

Therefore, the gut bacterial pathway of adenosine ribonucleotide de novo biosynthesis influences obesity via the lactic acid production mechanism. In contrast, the mechanisms underlying AGA are closely tied to cellular metabolism and signal transduction. Consequently, the effects of this pathway on obesity and AGA differ mechanistically. To address obesity accompanied by AGA, interventions targeting the gut bacterial pathway of adenosine ribonucleotide de novo biosynthesis should not only inhibit lactic acid formation but also activate K^ATP^ channels.

## 5. Conclusions

By employing a bidirectional MR analysis to investigate the relationships among the gut microbiome, obesity, and AGA, it was found that although obese individuals are more susceptible to AGA, there is no direct causal relationship between obesity and AGA. Two distinct gut bacterial pathways are implicated in both obesity and AGA. Inhibiting the abundance of the sulfoglycolysis pathway can mitigate obesity and improve AGA through a shared reaction mechanism. Conversely, the adenosine ribonucleotide de novo biosynthesis pathway exhibits different mechanisms for influencing obesity and AGA. Nonetheless, both pathways can serve as potential therapeutic targets for addressing obesity accompanied by AGA, offering a novel treatment strategy for obese individuals with AGA.

## 6. Limitations

The dataset utilized in this study was exclusively sourced from the European population, featuring a relatively limited number of case samples, particularly among obese individuals. Nevertheless, during the data screening process, methods such as a sensitivity analysis were employed to ensure that the selected dataset retained high-quality genotype characteristics, thereby mitigating the limitations associated with the small sample size. While the single-ethnicity composition may constrain the extrapolation of the study findings to non-European populations, the conclusions drawn here remain robustly reliable within the European context. Furthermore, considering the higher prevalence of obesity-associated AGA in Europeans compared to other ethnic groups, the study’s conclusions retain substantial relevance. It is important to highlight that the absence of phenotypic classification information, such as gender, ethnicity, and dietary patterns, in the current dataset could potentially influence the results. Consequently, future investigations should prioritize incorporating broader phenotypic data to enhance the comprehensiveness and precision of the research outcomes.

## Figures and Tables

**Table 1:** The bidirectional MR analysis revealed the association between 17 specific gut microbiomes and obesity-associated AGA.

Forward MR Analysis							Reserve MR Analysis
Exposure	Outcome	SNPs	OR (95% CI)	*P*_IVW_	*P*_Heterogeneity_	*P*_pleiotropy_	*P*_IVW_
PWY-7456: Mannan Degradation	Obesity	7	0.921 (0.818–1.038)	0.176	0.580	0.897	0.852
PWY-5989: Stearate Biosynthesis II (Bacteria and Plants)	Obesity	8	0.975 (0.872–1.091)	0.661	0.509	0.110	0.482
s. *Eubacterium eligens*	Obesity	13	0.961 (0.854–1.082)	0.512	0.038	0.319	0.873
PWY-5505: L-Glutamate and L-Glutamine Biosynthesis	Obesity	16	1.008 (0.931–1.093)	0.839	0.127	0.186	0.437
PWY-5791: 1,4-Dihydroxy-2-naphthoate Biosynthesis II (Plants)	Obesity	16	0.999 (0.930–1.074)	0.988	0.102	0.245	0.133
s. *Clostridium leptum*	Obesity	7	1.037 (0.960–1.120)	0.356	0.805	0.464	0.274
PWY-7446: Sulfoglycolysis	Obesity	10	1.066 (1.016–1.119)	0.009	0.669	0.715	0.464
s. *Bifidobacterium bifidum*	Obesity	16	1.030 (0.985–1.076)	0.191	0.744	0.692	0.641
PWY-5850: Superpathway of Menaquinol-6 Biosynthesis I	Obesity	11	1.001 (0.947–1.058)	0.971	0.192	0.577	0.073
PWY-5971: Palmitate Biosynthesis II (Bacteria and Plants)	Obesity	14	0.975 (0.872–1.091)	0.661	0.072	0.065	0.923
PWY-6700: Queuosine Biosynthesis	Obesity	16	1.019 (0.933–1.113)	0.678	0.555	0.489	0.433
s. *Bacteroides xylanisolvens*	Obesity	13	0.951 (0.870–1.039)	0.268	0.221	0.728	0.615
PWY-7219: Adenosine Ribonucleotide De Novo Biosynthesis	Obesity	13	1.115 (1.012–1.229)	0.028	0.935	0.831	0.432
g. *Barnesiella*	Obesity	14	1.078 (0.990–1.173)	0.082	0.405	0.122	0.297
s. *Barnesiella intestinihominis*	Obesity	13	1.076 (0.984–1.176)	0.110	0.373	0.177	0.295
PWY-5188: Tetrapyrrole Biosynthesis I (from Glutamate)	Obesity	11	1.030 (0.934–1.137)	0.553	0.693	0.955	0.811
PWY-5695: Urate Biosynthesis/Inosine-5′-Phosphate Degradation	Obesity	11	0.999 (0.889–1.122)	0.984	0.282	0.356	0.778

**Table 2:** The bidirectional MR analysis of the causal relationship between obesity and AGA.

Forward MR Analysis							Reserve MR Analysis
Exposure	Outcome	SNPs	OR (95% CI)	*P*_IVW_	*P*_Heterogeneity_	*P*_pleiotropy_	*P*_IVW_
Obesity	AGA	20	1.050 (0.994~1.110)	0.08	0.181	0.534	0.124

## Data Availability

The GWAS summary data used in this study were sourced from the GWAS Catalog, a publicly available, manually curated resource of all published GWAS and association results. The GWAS Catalog is collaboratively produced and maintained by the Human Genome Research Institute (NHGRI) and the European Bioinformatics Institute (EMBL-EBI). The catalog is accessible at https://www.ebi.ac.uk/gwas/ (accessed on 1 Octorber 2024).
